# Diminished accuracy of biomarkers of fibrosis in low replicative chronic hepatitis B

**DOI:** 10.1186/s12876-017-0658-x

**Published:** 2017-08-25

**Authors:** Faisal M. Sanai, Taha Farah, Khalid Albeladi, Faisal Batwa, Yaser Dahlan, Mohammed A. Babatin, Hamad Al-Ashgar, Hadeel AlMana, Khaled S. Alsaad, Khalid AlSwat, Abdulrahman Aljumah, Ibrahim H. AlTraif, Bahaa E. Kailani, Khalid I. Bzeizi

**Affiliations:** 1 Liver Disease Research Center, King Saud University, Riyadh, Saudi Arabia; 2 Department of Gastroenterology, Prince Sultan Military Medical City, Riyadh, Saudi Arabia; 3 Division of Gastroenterology, Department of Medicine, King Fahad Hospital, Jeddah, Saudi Arabia; 4 Division of Gastroenterology, Department of Medicine, King Faisal Specialist Hospital & Research Center, Riyadh, Saudi Arabia; 5 Department of Pathology, King Faisal Specialist Hospital & Research Center, Riyadh, Saudi Arabia; 6 Department of Pathology, King Abdulaziz Medical City, Riyadh, Saudi Arabia; 7 Hepatobiliary Sciences & Liver Transplantation, King Abdulaziz Medical City, Riyadh, Saudi Arabia; 8 Gastroenterology Unit, Department of Medicine, King Abdulaziz Medical City, PO Box: 9515, Jeddah, 21423 Saudi Arabia; 9 King Saud bin Abdulaziz University for Health Sciences, Riyadh, Saudi Arabia; 10 Gastroenterology Unit, Department of Medicine, King Fahad Armed Forces Hospital, Jeddah, Saudi Arabia

**Keywords:** Hepatitis B, Fibrosis, Biomarkers, APRI

## Abstract

**Background:**

We evaluated the diagnostic accuracy of aspartate aminotransferase (AST)-to-platelet ratio index (APRI), fibrosis-4 index (FIB-4), AST/alanine aminotransferase (ALT) ratio (AAR), and age-platelet index (API) for significant fibrosis (Metavir F2–4) in low-replicative (HBV DNA <20,000 IU/mL) chronic hepatitis B virus (HBV) patients.

**Methods:**

The sensitivity, specificity, and area under the receiver-operating characteristic curve (AUROC) of HBeAg-negative, low-replicative (*n* = 213) and high-replicative (HBV DNA ≥20,000 IU/mL, *n* = 153) patients was assessed.

**Results:**

Overall, 113 patients (30.9%) had F2–4 fibrosis. Of the low and high-replicative patients, 40 (18.8%) and 73 (47.7%) had F2–4, respectively (*P* < 0.0001). APRI ≥0.5 less frequently identified F2–4 fibrosis in low vs. high-replicative patients (48.7% vs. 69.6%, *P* = 0.032) and AAR identified it more frequently in low-replicative patients (37.5% vs. 19.4%, *P* = 0.037). FIB-4 and API were not different (*P* > 0.05) for identifying F2–4 fibrosis in low and high-replicative patients. Higher specificities were seen at the lowest cut-offs in low vs. high-replicative states for APRI (≥0.5, 98% vs. 68.9%), AAR (84.3% vs. 76.6%), FIB-4 (≥1.45, 97.5% vs. 87.8%) and API (>4, 94.8% vs. 93.8%). At ROC-defined thresholds, APRI (≥0.33), AAR (≥0.93), FIB-4 (≥0.70) and API (>2) showed greater AUROCs for F2–4 diagnosis in low replicative (0.80, 0.62, 0.81 and 0.71, respectively) vs. high-replicative patients (0.73, 0.52, 0.67 and 0.69, respectively).

**Conclusion:**

All 4 biomarkers in both, low and high-replicative HBV demonstrate modest accuracy for fibrosis diagnosis at conventional cut-offs. Lowering the cut-offs may increase the diagnostic relevance of these biomarkers, particularly for APRI and FIB-4 in low-replicative disease.

**Electronic supplementary material:**

The online version of this article (doi:10.1186/s12876-017-0658-x) contains supplementary material, which is available to authorized users.

## Background

Assessment of histological disease is of immense importance in managing chronic hepatitis B virus (HBV) patients. Treatment is strongly recommended in patients with significant fibrosis (for instance, METAVIR score F2–4). In the absence of liver biopsy, treatment decisions are based on the surrogate markers of histological disease, namely, elevated alanine aminotransferase (ALT) and high HBV DNA levels [1, 2].

Significant fibrosis in patients with low viremia may occur, with reports of progression to cirrhosis [3]. We have previously shown that F2–4 fibrosis occurs in about 18% of patients with HBV DNA <20,000 IU/mL [4]. Thus, since fibrosis occurs in a minority of patients with ‘low’ viremia levels, it becomes essential to identify its predictors, prior to undertaking liver biopsy. Furthermore, a single biopsy does not measure the dynamic nature of liver fibrosis [5].

Of the several combined biomarkers of fibrosis, FIB-4 (based on age, ALT, aspartate aminotransferase [AST] and platelet count), AST-to-platelet ratio index (APRI), AST/ALT ratio (AAR), and age-platelet index (API) have been well studied. Studies have suggested that these markers may be useful for predicting fibrosis and cirrhosis [6]. However, most validations occurred under controlled conditions and have shown sensitivity and specificity at the extreme stages of liver fibrosis. Additionally, previous studies have not evaluated these combination biomarkers in the context of low HBV DNA levels.

In this study, we aimed to evaluate the diagnostic performance of serum biomarkers in identifying significant fibrosis in an unselected cohort of HBeAg-negative, low-replicative, chronic hepatitis B patients.

## Methods

### Study patients

Consecutive patients were included by a retrospective search of clinical records and hospital databases for the period between January 2006 and January 2012. All data was anonymized and de-identified prior to analysis. The patients were included from four centres in Saudi Arabia. The institutional review boards of all centres (Retrospective Research Committee, Institutional Review Board, King Abdullah International Medical Research Centre; Research Advisory Council, King Faisal Specialist Hospital and Research Centre; Research and Ethics Committee, Prince Sultan Military Medical City; Central Institutional Review Board; Ministry of Health) approved the study. Eligible patients for the analysis had detectable HBsAg >6 months prior to inclusion in the study. All patients were HBeAg-negative and were between 16 to 80 years of age.

The exclusion criteria were: (i) co-infection with hepatitis C, delta virus or HIV; (ii) superimposed with other liver diseases; (iii) hepatotoxic medications in the preceding 3 months; (iv) previous immunosuppressive or antiviral therapy; (v) decompensated cirrhosis with a Child-Pugh score > 6, or evidence of portal hypertension, variceal bleeding, laboratory findings of a platelet count <100 (10^9^/L), an international normalized ratio ≥ 1.3; (vii) creatinine >135 μmol/L (viii) presence of hepato-biliary malignancy; (ix) alcohol consumption >20 g/day; and (x) organ transplantation.

### Study design

A minimum of three recordings of liver biochemistry for patients with normal levels and two recordings for those with elevated levels were required. The median number of ALT tests performed pre liver biopsy was 5 (interquartile range [IQR] 4–8), and these were sampled over a median period of 21 months (IQR 8–46). Serum biochemical and haematological values prior to the liver biopsy were recorded. Routine liver biochemical tests were performed using commercially available autoanalysers and hepatitis serological markers were assayed using commercially available enzyme-linked immunoassays. Patients with HBV DNA <20,000 IU/mL were classified as low-replicative while those with HBV DNA ≥20,000 IU/mL were classified as high-replicative. A minimum of three HBV DNA recordings was required and the reference value utilized for analysis was based on the pre biopsy level. Serum HBV DNA level was expressed in IU/mL (1 IU/mL = 5.6 copies/mL) [7]. Quantitative HBV DNA levels were measured by a COBAS TaqMan System (Roche Diagnostics, Indianapolis, IN, USA), which has a lower detection limit of 15 IU/mL, or Abbott Real-Time HBV assay (Abbott Molecular, Inc., Des Plaines, IL, USA), with a lower detection limit of 10 IU/mL. The median duration between the first and pre liver biopsy HBV DNA was 12 months (IQR 6–20).

### Measurement of noninvasive biomarkers

Patient data included age (at time of liver biopsy), ALT and AST levels (upper limit of normal [ULN] values that were specific to the laboratory performing the test) and platelet counts. APRI, FIB-4 and AST/ALT ratio were calculated based on laboratory results from sera collected within 3 months of the liver biopsy using the following formulas:

$$ {\displaystyle \begin{array}{l}\mathrm{FIB}\hbox{-} 4=\frac{\mathrm{Age}\ \left(\mathrm{years}\right)\times \mathrm{AST}\ \left(\mathrm{U}/\mathrm{L}\right)}{\mathrm{Platelet}\  \mathrm{count}\ \left({10}^9/\mathrm{L}\right)\times {\left[\mathrm{ALT}\ \left(\mathrm{U}/\mathrm{L}\right)\right]}^{1/2}}\kern2.28em \begin{array}{c}\hfill \mathrm{AAR}=\mathrm{AST}/\mathrm{ALT}\hfill \\ {}\hfill \hfill \end{array}\\ {}\mathrm{APRI}=\frac{\mathrm{AST}\ \left(/\mathrm{ULN}\right)}{\mathrm{Platelet}\  \mathrm{count}\ \left({10}^9/\mathrm{L}\right)}\times 100\end{array}} $$

API: Age (years): < 30 = 0; 30–39 = 1; 40–49 = 2; 50–59 = 3; 60–69 = 4; ≥70 = 5. Platelet count: >225 = 0; 200–224 = 1; 175–199 = 2; 150–174 = 3; 125–149 = 4; <125 = 5. AP index is the sum of the above (possible value 0–10).

### Liver histology

All patients included in this study had liver biopsies stained with hematoxylin and eosin for morphological evaluation and reticulin for the assessment of fibrosis. Only liver specimens with more than nine portal tracts were considered sufficient. All specimens were centrally assessed and scored according to the METAVIR scoring system [8] by two experienced hepatopathologists who were blinded to all clinical information. Fibrosis scores of F0–1 were defined as minimal/mild, F2–4 as significant.

### Statistical analyses

Quantitative variables were expressed as the mean ± standard deviation, and categorical variables as frequencies and proportions. The unpaired student’s t-test was used to compare between the means or log means of the variable if the variable was normally or not normally distributed, respectively. The Chi-square or Fishers’ exact tests were used to compare frequencies and proportions in categorical variables, as appropriate. The sensitivity, specificity, positive likelihood ratio (LR+), and negative likelihood ratio (LR-) of points on curve were calculated to obtain the optimal cut-off point to discriminate F2–4 with noninvasive biomarkers. The area under receiver operating characteristics (AUROC) curve was used to assess the overall diagnostic value of the biomarkers at different cut-offs that predicted fibrosis. All of the tests of significance were 2-tailed and a *P*-value of <0.05 was considered statistically significant. Statistical Package for Social Sciences (*SPSS, version* 17.0; Chicago, IL, USA) and MedCalc (MedCalc Software, Inc., Mariakerke, Belgium) were used for data analysis.

## Results

### Baseline characteristics of patients

A total of 366 patients were included in this analysis and of these 213 (58.2%) had HBV DNA < 20,000 IU/mL, and 153 (41.8%) had HBV DNA ≥ 20,000 IU/mL. The mean age was 38.6 ± 11.8 years and males constituted 272 (74.3%). In the overall cohort, F2–4 fibrosis was seen in 113 (30.9%) patients of whom 20 (5.5%) had F4 fibrosis. Of the low-replicative (HBV DNA <20,000 IU/mL) and high-replicative (≥20,000 IU/mL) patients, 40 (18.8%) and 73 (47.7%) had F2–4, respectively (*P* < 0.0001, Table 1). Low replicative patients were more obese compared to high-replicative ones (*P* = 0.002) and more likely to have normal ALT (55.4% vs. 19.6%, *P* < 0.0001). Mean biomarker scores between low-replicative and high-replicative patients were not significantly different (*P* > 0.05), however the mean scores were significantly higher for all biomarkers for F2–4 (compared to F0–1, *P* < 0.05, data not shown). Furthermore, all four biomarkers more frequently identified F2–4 fibrosis in both, low and high-replicative patients (*P* < 0.05, Additional file 1: Table S1).

**Table 1:** Baseline Characteristics of patients in relation to HBV DNA levels

Parameters	HBV DNA < 20,000 IU/mL (*n* = 213)	HBV DNA > 20,000 IU/mL (*n* = 153)	*P* value
Age (years)	38.2 ± 11.7	39.1 ± 12.0	0.485
Male gender (%)	156 (73.2)	116 (75.8)	0.578
BMI (Kg/m^2^)	29.5 ± 10.4	26.9 ± 4.9	0.002
Diabetes mellitus (%)	30 (14.1)	20 (13.1)	0.781
Hyperlipidemia (%)	41 (19.2)	31 (20.3)	0.810
HBV DNA Log_10_	3.3 ± 0.7	6.1 ± 1.3	< 0.0001
Platelets (10^9^)	255.3 ± 68.9	232.2 ± 61.8	0.001
Elevated AST (%)	41 (20.0)	80 (54.1)	< 0.0001
Normal ALT (%)	118 (55.4)	30 (19.6)	< 0.0001
Elevated ALT (%)	95 (44.6)	123 (80.4)	
Fibrosis (F2–4, %)	40 (18.8)	73 (47.7)	< 0.0001

Data expressed as mean ± standard deviation or n (%) as appropriate. n, number. *AST* aspartate aminotransferase, *ALT* alanine aminotransferase ratio, *BMI* body mass index, *HBV* hepatitis B virus

Overall, there was an association between fibrosis stage and APRI, FIB-4 and API (*P* < 0.05 for all). For each successive fibrosis stage from F0 to F4, significantly higher APRI, FIB-4 and API values were seen (*P* < 0.0001 for all, Table 2). There was no difference between successive fibrosis stages by AAR (*P* = 0.445).

**Table 2:** Mean scores of the four biomarkers in increasing stages (Metavir) of liver fibrosis

Fibrosis Stage	APRI (95% CI)	*P* value	AAR (95% CI)	*P* value	FIB-4 (95% CI)	*P* value	API (95% CI)	*P* value
STAGE 0	0.32 (0.27–0.38)	< 0.0001	0.77 (0.31–0.42)	0.445	0.66 (0.54–0.78)	< 0.0001	1.58 (1.22–1.94)	< 0.0001
STAGE 1	0.38 (0.31–0.45)		0.65 (0.69–0.83)		0.71 (0.63–0.78)		1.74 (1.52–1.96)	
STAGE 2	0.71 (0.52–0.90)		0.68 (0.60–0.77)		1.08 (0.88–1.29)		2.59 (2.11–3.07)	
STAGE 3	0.88 (0.66–1.09)		0.86 (0.58–1.14)		1.66 (1.06–2.25)		3.56 (2.68–4.44)	
STAGE 4	1.13 (0.51–1.76)		0.96 (0.68–1.24)		2.52 (1.49–3.55)		5.10 (3.93–6.27)	

*AST* aspartate aminotransferase, *APRI* AST-to-platelet ratio index, *AAR* AST/alanine aminotransferase ratio, *API* age-platelet index

### Sensitivity and specificity of biomarkers in relation to HBV DNA levels

For high-replicative patients, an APRI ≥0.5 more frequently identified F2–4 fibrosis (69.6%) as compared to those with low-replication (48.7%, *P* = 0.032). Other APRI cut-offs were not significantly different between the two groups (Table 3). On the other hand, AAR >1.0 less frequently identified F2–4 fibrosis in high-replicative patients (19.4 vs. 37.5%, *P* = 0.037). None of the cut-offs for FIB-4 or API differentiated F2–4 vs. F0–1 fibrosis between low and high-replicative patients. The specificity for F2–4 fibrosis at the lowest cut-offs was consistently greater in low compared to high-replicative states for APRI (98.0% vs. 68.9%, respectively), AAR (84.3% vs. 76.6%, respectively), FIB-4 (97.5 vs. 87.8%, respectively) and API (94.8% vs. 93.8%, respectively). This pattern remained similar for higher cut-offs (Table 4).

**Table 3:** Diagnosis of significant fibrosis (F2–4) in low and high replication HBV patients based on the four biomarkers

Fibrosis Biomarker	Total (*n* = 113)	HBV DNA < 20,000 IU/mL (*n* = 40)	HBV DNA > 20,000 IU/mL (*n* = 73)	*P* value
AST-Platelet Ratio Index (APRI)				
≥0.5	67 (62.0)	19 (48.7)	48 (69.6)	0.032
≥0.7	46 (42.6)	13 (32.3)	33 (47.8)	0.143
≥1.0	26 (24.1)	7 (17.9)	19 (27.5)	0.263
≥1.5	11 (10.2)	2 (5.1)	9 (13.0)	0.191
AST/ALT Ratio (AAR)				
>1.0	29 (25.9)	15 (37.5)	14 (19.4)	0.037
FIB-4				
≥1.45	35 (32.4)	14 (35.9)	21 (30.4)	0.560
≥2.0	25 (23.1)	9 (23.1)	16 (23.2)	0.999
≥3.25	10 (9.3)	6 (15.4)	4 (5.8)	0.164
Age-Platelet Index (API)				
>4	34 (30.1)	15 (37.5)	19 (26.0)	0.204
>5	21 (8.6)	9 (22.5)	12 (6.4)	0.428
>6	12 (10.6)	6 (15.0)	6 (8.2)	0.263
>7	5 (4.4)	3 (7.5)	2 (2.7)	0.344

Data expressed as n (%). *N* number, *AST* aspartate aminotransferase, *ALT* alanine aminotransferase ratio

**Table 4:** Sensitivity, specificity and AUROCs of the four different biomarkers in patients with low and high-replicative HBV at different cutoff values in identifying significant fibrosis (F2–4)

Biomarker Cut-off	HBV DNA ≤ 20,000 IU/mL			HBV DNA ≥ 20,000 IU/mL		
	Sensitivity (%, 95% CI)	Specificity (%, 95% CI)	AUROC (%, 95% CI)	Sensitivity (%, 95% CI)	Specificity (%, 95% CI)	AUROC (%, 95% CI)
	AST-Platelet Ratio Index (APRI)			AST-Platelet Ratio Index (APRI)		
≥0.5	48.7 (32.4–65.2)	98.0 (91.2–99.5)	0.70 (0.63–0.77)	69.6 (57.3–80.1)	68.9 (57.1–79.2)	0.69 (0.61–0.77)
≥0.7	33.3 (19.1–50.2)	98.1 (94.5–99.6)	0.66 (0.59–0.72)	47.8 (35.7–60.2)	77.0 (65.8–86.0)	0.62 (0.54–0.70)
≥1.0	17.9 (7.5–33.5)^a^	99.4 (96.5–100.0)	0.59 (0.51–0.66)	27.5 (17.5–39.6)	91.9 (83.2–97.0)	0.60 (0.51–0.68)
≥1.5	5.1 (0.6–17.3)^a^	100.0 (97.7–100.0)	0.53 (0.45–0.60)	13.0 (6.1–23.3)^a^	95.9 (88.6–99.2)	0.54 (0.46–0.63)
	AST/ALT Ratio (AAR)			AST/ALT Ratio (AAR)		
>1.0	37.5 (22.7–54.2)	84.3 (77.9–89.5)	0.61 (0.54–0.68)	19.4 (11.1–30.5)	76.6 (65.6–85.5)	0.48 (0.40–0.56)
	FIB-4			FIB-4		
≥1.45	35.9 (21.2–52.8)	97.5 (93.7–99.3)	0.67 (0.60–0.73)	30.4 (19.9–42.7)	87.8 (78.2–94.3)	0.59 (0.51–0.67)
≥2.0	23.1 (11.1–39.3)	98.7 (95.5–99.9)	0.61 (0.54–0.68)	23.2 (13.9–34.9)	91.9 (83.2–97.0)	0.58 (0.49–0.66)
≥3.25	15.4 (5.9–30.5)^a^	100.0 (97.7–100.0)	0.58 (0.50–0.65)	5.8 (1.6–14.2)^a^	97.3 (90.6–99.7)	0.52 (0.43–0.60)
	Age-Platelet Index (API)			Age-Platelet Index (API)		
>4	37.5 (22.7–54.2)	94.8 (90.3–97.6)	0.66 (0.59–0.72)	15.6 (7.7–26.9)	93.8 (86.0–97.9)	0.55 (0.46–0.63)
>5	22.5 (10.8–38.5)	97.1 (93.4–99.1)	0.60 (0.53–0.66)	16.4 (8.8–26.9)	96.3 (89.4–99.2)	0.56 (0.48–0.64)
>6	15.0 (5.7–29.8)^a^	100.0 (97.9–100.0)	0.58 (0.51–0.64)	8.2 (3.0–17.0)^a^	98.8 (93.2–99.9)	0.53 (0.45–0.59)
>7	7.5 (1.6–20.4)^a^	100.0 (97.9–100.0)	0.54 (0.47–0.61)	2.7 (0.3–9.6)^a^	98.8 (93.2–99.9)	0.51 (0.43–0.59)

*AST* aspartate aminotransferase, *ALT* alanine aminotransferase ratio, *HBV* Hepatitis B virus, *AUROC* Area under the receiver-operating characteristic. *numbers low in cells, analysis to be interpreted with caution

### Performance characteristics of biomarkers

The overall sensitivity, specificity, LR+, LR- and AUROCs of the four biomarkers at different cut-offs for the diagnosis of F2–4 fibrosis are shown in Additional file 2: Table S2. APRI demonstrated similar AUROCs for low and high-replicative states at all cut-offs (Table 4). However, higher AUROCs for F2–4 diagnosis were observed for low- compared to high-replicative state with AAR (0.61 vs. 0.48), FIB-4 (0.67 vs. 0.59) and API (0.66 vs. 0.55). Additionally, the AUROCs were serially lower across all biomarkers for both low and high-replicative states with incremental increase in the cut-off levels.

**Fig. 1:**
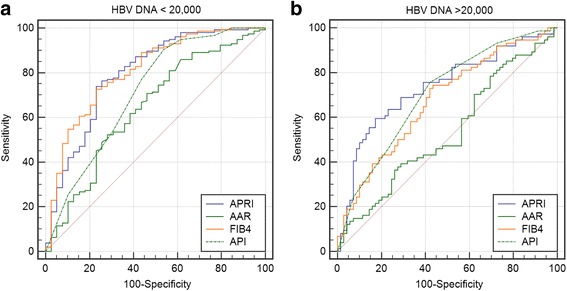
**a** Receiver operating characteristics curve of the best compromise sensitivity-specificity of AST (aspartate aminotransferase)-platelet ratio index (APRI), AST/alanine aminotransferase ratio (AAR), FIB-4 index and age-platelet index (API) for identifying fibrosis score F2–4 in **low-replicative** HBV patients. In patients meeting these cut-offs, the sensitivity for APRI (≥0.33) is 76.4% and the specificity is 74.4% (AUROC, 0.80; 95% CI: 0.73–0.85; *P* < 0.0001), for AAR (≥0.93) the sensitivity is 73.5% and the specificity 47.5% (AUROC, 0.62; 95% CI: 0.55–0.69; *P* = 0.023), for FIB-4 (≥0.70) the sensitivity is 72.9% and specificity is 76.9% (AUROC, 0.81; 95% CI: 0.75–0.86; *P* < 0.0001), and for API (>2) the sensitivity is 75.1% and specificity is 55.0% (AUROC, 0.71; 95% CI: 0.64–0.77; *P* < 0.0001). **b** Receiver operating characteristics curve of the best compromise sensitivity-specificity of AST (aspartate aminotransferase)-platelet ratio index (APRI), AST/alanine aminotransferase ratio (AAR), FIB-4 index and age-platelet index (API) for identifying fibrosis score F2–4 in **high-replicative** HBV patients. In patients meeting these cut-offs, the sensitivity for APRI (≥0.33) is 52.7% and the specificity is 87.0% (AUROC, 0.73; 95% CI: 0.65–0.80; *P* < 0.0001), for AAR (≥0.91) the sensitivity is 24.7% and the specificity 79.2% (AUROC, 0.52; 95% CI: 0.44–0.60; *P* = 0.643), for FIB-4 (≥0.70) the sensitivity is 58.1% and specificity is 66.7% (AUROC, 0.67; 95% CI: 0.59–0.75; *P* = 0.0001), and for API (>2) the sensitivity is 75.0% and specificity is 56.2% (AUROC, 0.69; 95% CI: 0.61–0.76; *P* < 0.0001)

Figure 1a depicts the ROC curve analysis for optimum cut-offs with the best compromise sensitivity-specificity for distinguishing F2–4 for the four biomarkers in low-replicative state. At the ROC-derived optimum cut-offs, the sensitivity for APRI (≥0.33) is 76.4% and the specificity is 74.4% (AUROC, 0.80; 95% CI: 0.73–0.85; *P* < 0.0001), for AAR (≥0.93) the sensitivity is 73.5% and the specificity 47.5% (AUROC, 0.62; 95% CI: 0.55–0.69; *P* = 0.023), for FIB-4 (≥0.70) the sensitivity is 72.9% and specificity is 76.9% (AUROC, 0.81; 95% CI: 0.75–0.86; *P* < 0.0001), and for API (>2) the sensitivity is 75.1% and specificity is 55.0% (AUROC, 0.71; 95% CI: 0.64–0.77; *P* < 0.0001). Figure 1b depicts the ROC curve analysis in high-replicative patients. In patients meeting these optimum cut-offs, the sensitivity for APRI (≥0.33) is 52.7% and the specificity is 87.0% (AUROC, 0.73; 95% CI: 0.65–0.80; *P* < 0.0001), for AAR (≥0.91) the sensitivity is 24.7% and the specificity 79.2% (AUROC, 0.52; 95% CI: 0.44–0.60; *P* = 0.643), for FIB-4 (≥0.70) the sensitivity is 58.1% and specificity is 66.7% (AUROC, 0.67; 95% CI: 0.59–0.75; *P* = 0.0001), and for API (>2) the sensitivity is 75.0% and specificity is 56.2% (AUROC, 0.69; 95% CI: 0.61–0.76; *P* < 0.0001).

## Discussion

Among the various markers available for evaluating HBV-related fibrosis, the ones utilized in this analysis are the most commonly studied, albeit not specifically in the context of low viremia levels. We have shown that APRI, FIB-4, AAR and API frequently identify F2–4 fibrosis, regardless of the HBV DNA levels. These findings are in concurrence with other studies dealing with chronic HBV in general that show a moderate utility in distinguishing F2–4 from F0–1 fibrosis. However, our analysis also demonstrates significant differences in the detection ability between patients with low and high-replicative states. Clearly, APRI seems to perform better in high-replicative patients (with only the cut-off ≥0.5 reaching statistical significance). However, the other biomarkers appear to perform better in patients with low-replicative states, detecting fibrosis more frequently than in those with high replication.

According to our analysis, at their lowest conventional cut-off points, the AUROCs of all the biomarkers were of low diagnostic value, with only APRI (AUROC 0.62) reaching statistical significance. At a cut-off point of 0.5, the higher sensitivity of APRI in high-replicative states was offset by a low specificity (69%), while those with low replication exhibited a high specificity of 98%. Similar results were seen in the sensitivity and specificity profile of the other biomarkers. However, despite these higher specificities, the overall accuracy of these biomarkers in diagnosing fibrosis remains poor in both groups of patients (<0.70 for all biomarkers at all conventional cut-offs), being far below the standard of diagnostic relevance (typically >0.80).

Interestingly, AAR appears better at distinguishing fibrosis in those with low replication. However, overall AAR only detects a quarter of the patients with F2–4 fibrosis with a diagnostic accuracy of 0.54. Thus, its poor performance as a predictor of fibrosis makes it unsuitable for use in clinical settings. Moreover, these observations can also be extended to FIB-4 and API where significant fibrosis was missed in more than two-thirds of the overall cohort of patients at their lowest cut-offs, thereby diminishing the clinical relevance of these findings. Nonetheless, when analysing patients at ROC-derived optimal cut-offs, all four biomarkers performed consistently better in the low-replicative state, suggesting greater diagnostic utility of these biomarkers in this category of patients. Our analysis shows that by lowering the cut-offs for APRI and FIB-4 a high diagnostic accuracy (>0.80) may be achieved in distinguishing F2–4 from F0–1 fibrosis in patients with low-replicative HBV.

The sensitivity levels of the biomarkers in identifying fibrosis in our study are somewhat lower (i.e. high number of patients with fibrosis incorrectly identified as being without) than that seen in other studies. In a recent meta-analysis [6], the summary sensitivities of APRI at cut-off levels of 0.5, 0.7, 1.0 and 1.5 in identifying F2–4 fibrosis were 70, 63.5, 40 and 34%, respectively, compared to the sensitivities in our analysis of 62, 43, 24 and 10%, respectively. Similar differences were also seen in the summary sensitivities of FIB-4. These differences must be seen in the context of the patient population being studied, where in previous studies the HBV population was mostly comprised of patients with high viral replication and elevated ALT levels. In contrast, our study population bears a close resemblance to real-life HBeAg-negative presentation, where a large number of patients had low replication and/or normal ALT. These consecutive patients also included those with ‘high’ replication and normal ALT, and ‘low’ replication with elevated ALT, eventually serving as a robust representative sample of HBeAg-negative patients. Thus, it is likely that the sensitivities and specificities shown in our analysis are closer to a real-life population, and the findings more representative.

The impact of elevated ALT and AST in our cohort could be more confounding than is apparent at face value. In most studies, patients with HBV who undergo liver biopsy are those that have high viral replication. However, in a more natural selection of HBV infected population, as in our cohort, liver enzyme elevation may be related to non-HBV factors such as steatosis, which in turn does not impact on development of fibrosis [4, 9, 10]. As such, this elevation of ALT and AST levels may cause an “artefactual” increase in APRI, FIB-4 and AAR thereby increasing the false-positive rate (i.e. high number of patients without fibrosis incorrectly identified as having disease).

Our analysis must be viewed in the context of its limitations. First, although we relied on pre-biopsy ALT and AST levels, it has been shown that HBV patients may show enzyme fluctuations [3, 11, 12], and consequently some of these patients could feasibly be underscored, having dropped or increased the enzyme levels at the time of biopsy. Similarly, HBV DNA levels may also fluctuate over time, although fluctuations at 20,000 IU/mL are uncommon [13, 14]. In order to correct for such aberrations we categorized patients on the basis of the HBV DNA level recordings falling predominantly within the high or low replicative state. Overall, despite guideline-stratified thresholds, it remains a questionable approach to divide patients arbitrarily into low and high replicative states given the nature of HBV DNA fluctuations. Second, test accuracy can be ascertained when there is no selection bias in evaluating the reported outcome. Our population of HBV patients does not essentially represent an unselected cohort, given the impracticality of performing a liver biopsy in all HBeAg-negative patients. It is feasible that a proportion of these patients may have been biopsied as a consequence of subtle hints for the presence of fibrosis, particularly since patients who are not biopsied tend to have lower ALT and HBV DNA levels, thus escaping the trigger for a liver biopsy [14]. Clearly, these results must be validated in larger, unselected and properly stratified HBV-infected populations before generalizing these results. Despite these limitations, our study with a substantially large number of patients with low viral replication, offers a leading insight into a poorly appreciated disease state.

## Conclusion

In conclusion, our study shows a modest accuracy of commonly used biomarkers in the diagnosis of significant fibrosis. In low-replicative patients, at conventional cut-offs, APRI is less useful while AAR is more useful in detecting fibrosis as compared to high replication patients, although it detects only a small minority of patients with fibrosis. Lowering the cut-offs may increase the diagnostic relevance of these biomarkers, particularly for APRI and FIB-4 in low-replicative disease.

## Additional files

Additional file 1: Table S1. Comparison of the 4 biomarkers scores at various thresholds in low and high viremia levels between patients with minimal/mild (F0–1) and moderate-severe (F2–4) fibrosis. (DOCX 18 kb)

Additional file 2: Table S2 Sensitivity, specificity, positive and negative likelihood ratios of the four biomarkers at different cut-off values in identifying significant fibrosis (F2–4) in the overall cohort (*n* = 366) of patients. (DOCX 16 kb)

## Abbreviations

AFP: α-fetoprotein
ALT: Alanine aminotransferase
AST: Aspartate aminotransferase
BMI: Body mass index
HBeAg: Hepatitis B envelope antigen
HBsAg: Hepatitis B surface antigen
HBV: Hepatitis B virus
ULN: Upper limit of normal
