# Supplementary material for: Diminished accuracy of biomarkers of fibrosis in low replicative chronic hepatitis B
Source: BMC Gastroenterol. 2017 Aug 25;17:101. doi: 10.1186/s12876-017-0658-x (PMC5574089; doi:10.1186/s12876-017-0658-x)
Supplement: Supplementary file 1 — Comparison of the 4 biomarkers scores at various thresholds in low and high viremia levels between patients with minimal/mild (F0–1) and moderate-severe (F2–4) fibrosis. (DOCX 18 kb) [file 12876_2017_658_MOESM1_ESM.docx]

Table S1: Comparison of the 4 biomarkers scores at various thresholds in low and high viremia levels between patients with minimal/mild (F0-1) and moderate-severe (F2-4)

| **Fibrosis biomarker** | **HBV DNA <20,000 IU/mL** | | | **HBV DNA >20,000 IU/mL** | | |
| --- | --- | --- | --- | --- | --- | --- |
|  | F0-1 (n=173) | F2-4 (n=40) | *P* value | F0-1 (n=80) | F2-4 (n=73) | *P* value |
| **AST-Platelet Ratio Index (APRI)** | | | | | | |
| ≥0.5 | 13 (8.3) | 19 (48.7) | < 0.0001 | 23 (31.1) | 48 (69.6) | < 0.0001 |
| ≥0.7 | 3 (1.9) | 13 (33.3) | < 0.0001 | 17 (23.0) | 33 (47.8) | 0.003 |
| ≥1.0 | 1 (0.6) | 7 (17.9) | < 0.0001 | 6 (8.1) | 19 (27.5) | 0.004 |
| ≥1.5 | 0 (0.0) | 2 (5.1) | - | 3 (4.1) | 9 (13.0) | 0.071 |
| Mean | 0.28 ± 0.16 | 0.72 ± 0.92 | < 0.0001 | 0.53 ± 0.64 | 0.88 ± 0.76 | 0.004 |
| **AST/ALT Ratio (AAR)** | | | | | | |
| >1.0 | 26 (15.7) | 15 (37.5) | 0.002 | 18 (23.4) | 14 (19.4) | 0.690 |
| Mean | 0.78 ± 0.44 | 0.96 ± 0.54 | 0.028 | 0.72 ± 0.50 | 0.67 ± 0.44 | 0.508 |
| **FIB-4** | | | | | | |
| ≥1.45 | 4 (2.5) | 14 (35.9) | < 0.0001 | 9 (12.2) | 21 (30.4) | 0.008 |
| ≥2.0 | 2 (1.3) | 9 (23.1) | < 0.0001 | 6 (8.1) | 16 (23.2) | 0.019 |
| ≥3.25 | 0 (0.0) | 6 (15.4) | - | 2 (2.7) | 4 (5.8) | 0.429 |
| Mean | 0.63 ± 0.35 | 1.67 ± 1.76 | < 0.0001 | 0.84 ± 0.71 | 1.34 ± 1.09 | 0.001 |
| **Age-Platelet Index (API)** | | | | | | |
| >4 | 9 (5.2) | 15 (37.5) | < 0.0001 | 5 (6.2) | 19 (26.0) | 0.001 |
| >5 | 5 (2.9) | 9 (22.5) | < 0.0001 | 3 (3.8) | 12 (16.4) | 0.012 |
| >6 | 0 (0.0) | 6 (15.0) | - | 1 (1.2) | 6 (8.2) | 0.054 |
| >7 | 0 (0.0) | 3 (7.5) | - | 1 (1.2) | 2 (2.7) | 0.606 |
| Mean | 1.64 ± 1.48 | 3.45 ± 2.55 | < 0.0001 | 1.83 ± 1.61 | 3.16 ± 2.17 | < 0.0001 |

Data expressed as mean ± standard deviation or n (%) as appropriate. n; number. AST, aspartate aminotransferase;

ALT, alanine aminotransferase ratio
