# Supplementary material for: Diminished accuracy of biomarkers of fibrosis in low replicative chronic hepatitis B
Source: BMC Gastroenterol. 2017 Aug 25;17:101. doi: 10.1186/s12876-017-0658-x (PMC5574089; doi:10.1186/s12876-017-0658-x)
Supplement: Supplementary file 2 — Sensitivity, specificity, positive and negative likelihood ratios of the four biomarkers at different cut-off values in identifying significant fibrosis (F2–4) in the overall cohort (n = 366) of patients. (DOCX 16 kb) [file 12876_2017_658_MOESM2_ESM.docx]

Table S2: Sensitivity, specificity, positive and negative likelihood ratios of the four biomarkers at different cut-off values in identifying significant fibrosis (F2-4) in the overall cohort (n=366)

| **Biomarker cutoff** | **Sensitivity (95% CI)** | **Specificity (95% CI)** | **LR+ (95% CI)** | **LR- (95% CI)** | **AUROC (95 CI)** |
| --- | --- | --- | --- | --- | --- |
| **AST-Platelet Ratio Index (APRI)** | | | | | |
| ≥0.5 | 62.04 (52.19-71.20) | 84.42 (79.08-88.84) | 3.98 (2.85-5.56) | 0.45 (0.35-0.57) | 0.62 (0.52-0.71)* |
| ≥0.7 | 42.59 (33.13-52.47) | 91.34 (86.95-94.63) | 4.92 (3.07-7.89) | 0.63 (0.53-0.74) | 0.59 (0.47-0.71) |
| ≥1.0 | 24.07 (16.37-33.25) | 96.97 (93.86-98.77) | 7.94 (3.56-1.77) | 0.78 (0.70-0.87) | 0.58 (0.40-0.75) |
| ≥1.5 | 10.19 (5.20-17.49) | 98.70 (96.25-99.73) | 7.84 (2.23-2.75) | 0.91 (0.85-0.97) | 0.67 (0.37-0.89) |
| ≥0.31** | 85.19 (77.1-84.5) | 65.80 (59.30-71.90) | 2.49 (2.00-3.00) | 0.23 (0.10-0.40) | 0.80 (0.76-0.85)* |
| **AST/ALT Ratio (AAR)** | | | | | |
| >1.0 | 25.89 (18.08-35.03) | 81.89 (76.46-86.52) | 1.43 (0.95-2.16) | 0.91 (0.80-1.03) | 0.54 (0.42-0.66) |
| ≥0.38** | 79.46 (70.80-86.52) | 11.11 (7.50-15.81) | 0.89 (0.80-1.00) | 1.85 (1.10-3.10) | 0.50 (0.45-0.55) |
| **FIB-4** | | | | | |
| ≥1.45 | 32.41 (23.72-42.09) | 94.40 (90.61-96.98) | 5.78 (3.19-10.48) | 0.72 (0.63-0.82) | 0.60 (0.45-0.74) |
| ≥2.0 | 23.15 (15.57-32.25) | 96.55 (93.22-98.50) | 6.71 (3.13-14.39) | 0.80 (0.72-0.89) | 0.59 (0.41-0.76) |
| ≥3.25 | 9.26 (4.53-16.37) | 99.14 (96.92-99.90) | 10.74(2.39-48.18) | 0.92 (0.86-0.97) | 0.85 (0.54-0.98)* |
| ≥0.89** | 56.48 (46.62-66.01) | 83.62 (78.20-88.10) | 3.45 (2.50-4.80) | 0.52 (0.40-0.60) | 0.75 (0.70-0.79)* |
| **Age-Platelet Index (API)** | | | | | |
| >4 | 30.09 (21.82-39.43) | 94.47 (90.89-96.94) | 5.44 (3.04-9.73) | 0.74 (0.65-0.84) | 0.60 (0.45-0.74) |
| >5 | 18.58 (11.89-26.99) | 96.84 (93.86-98.63) | 5.88 (2.68-12.87) | 0.84 (0.77-0.92) | 0.71 (0.51-0.86)* |
| >6 | 10.62 (5.61-17.72) | 99.60 (97.82-99.99) | 26.86(3.54-204.1) | 0.90 (0.84-0.96) | - |
| >7 | 4.42 (1.45-10.02) | 99.60 (97.82-99.99) | 11.20(1.32-94.73) | 0.96 (0.92-1.00) | - |
| >2** | 55.75 (46.11-65.10) | 75.10 (69.33-80.32) | 2.24 (1.77-2.99) | 0.59 (0.50-0.70) | 0.70 (0.65-0.75)* |

Data expressed as n (%). N, number; AST, aspartate aminotransferase; ALT, alanine aminotransferase ratio. *P<0.05; **Receiver operator characteristic-derived optimal cutoff
